# Lipid droplet biogenesis from specialized ER subdomains

**DOI:** 10.15698/mic2020.08.727

**Published:** 2020-06-16

**Authors:** Vineet Choudhary, Roger Schneiter

**Affiliations:** 1 All India Institute of Medical Sciences (AIIMS), Department of Biotechnology, New Delhi, 110029, India.; 2 University of Fribourg, Department of Biology, 1700 Fribourg, Switzerland.

**Keywords:** ER subdomains, lipid droplet, seipin, Nem1, Yft2, Pex30, diacylglycerol

## Abstract

Lipid droplets (LDs) are cellular compartments dedicated to the storage of metabolic energy in the form of neutral lipids, commonly known as “fat”. The biogenesis of LDs takes place in the endoplasmic reticulum (ER), but its spatial and temporal organization is poorly understood. How exactly sites of LD formation are selected and the succession of proteins and lipids needed to mediate this process remains to be defined. In our current study we show that the yeast triacylglycerol (TAG)-synthases, Lro1 and Dga1 get recruited to discrete ER subdomains where they initiate TAG synthesis and hence LD formation (Choudhary *et al.* (2020), J Cell Biol). These ER subdomains are defined by yeast seipin, Fld1, and a regulator of diacylglycerol (DAG) production, Nem1. Both Fld1 and Nem1 are ER proteins which localize at contact sites between the ER and LDs. Interestingly, even in cells lacking LDs, Fld1 and Nem1 show punctate localization at ER subdomains independently of each other, but they are required together to recruit the TAG-synthases and hence create functional sites of LD biogenesis. Fld1/Nem1-containing ER subdomains recruit additional LD biogenesis factors, such as Yft2, Pex30, Pet10 and Erg6, and these membrane domains become enriched in DAG. In conclusion, Fld1 and Nem1 play a crucial role in defining ER subdomains for the recruitment of proteins and lipids needed to initiate LD biogenesis.

Lipid droplets (LDs) constitute a widely conserved fat storage compartment that plays a crucial role in cell physiology and metabolism. Growing evidence implicates LDs in many cellular processes, including the endoplasmic reticulum (ER) stress response, protein degradation, membrane trafficking and signal transduction, assembly of infectious viruses, and even for temporary storage of proteins. Defects in LD function in hepatocytes, macrophages and adipocytes leads to pathological conditions, including hepatic steatosis, cardiovascular diseases and obesity. LDs are composed of a core of neutral lipids, mainly triacylglycerols (TAG) and steryl esters (STE), surrounded by a monolayer of phospholipids. The LD surface harbors many lipid metabolic enzymes including lipases and acyltransferases, and structural proteins, such as perilipins (Pet10 in yeast). The biogenesis of LDs takes place in the ER membrane, where the enzymes that catalyze neutral lipid formation are located. The yeast genome encodes two TAG-synthesizing enzymes, Lro1 and Dga1, both convert DAG to TAG and therefore promote LD formation in the ER. TAG and STE are thought to accumulate within the ER bilayer, where they coalesce into lens-like structures. These neutral lipid lenses further grow in size and eventually emerge as LDs towards the cytoplasm, while staying connected to the ER (**Fig. 1**). Whether these neutral lipid lenses form randomly within the ER or at specific sites, however, remained to be defined.

**Figure 1:**
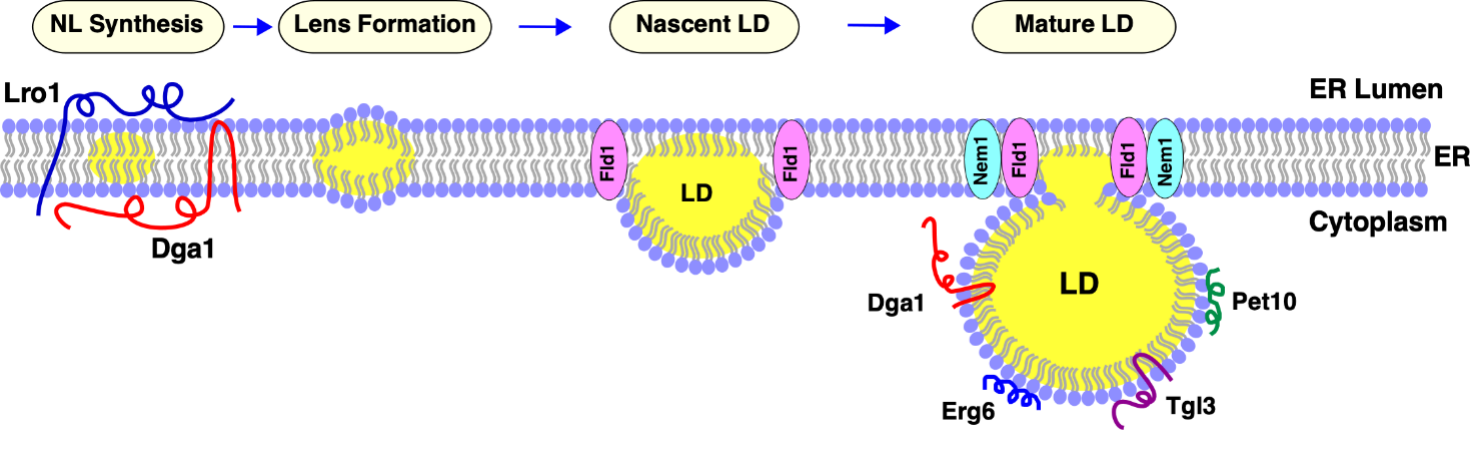
FIGURE 1: LD biogenesis in the ER. ER-localized triacylglycerol producing enzymes, Lro1, and Dga1 catalyze neutral lipid (NL, indicated in yellow) synthesis from opposite sides of the ER membrane. The NL then accumulate between the two-leaflets of the ER membrane leading to formation of lipid lenses. These NL lenses grow in size to become nascent LDs, which then emerge towards the cytoplasm where they further mature. Fld1 and Nem1 proteins show punctate localization at ER-LD contact sites. The acyltransferase Dga1, and the TAG lipase Tgl3 translocate onto the periphery of mature LDs. Finally, LD-marker proteins such as the perilipin ortholog Pet10 and the sterol biosynthetic enzyme Erg6 decorate the surface of the mature LD.

Several proteins have been implicated in LD formation, including seipin (Fld1 in yeast), lipin (Pah1 in yeast), fat-storage-inducing transmembrane protein, FIT2 (Yft2 and Scs3 in yeast) and the membrane shaping protein, Pex30. Seipin is an ER membrane protein that localizes to ER-LD contact sites and fulfils a key function in LD biogenesis. Mutations in human seipin severely impair fat deposition in adipocytes, resulting in a clinical feature known as lipodystrophy. The molecular function of seipin in LD formation has remained elusive, although recent studies suggest a structural and functional role – because it forms large oligomeric structures at the base of LDs that may couple lipid transfer between the ER and LDs. Lipin is a phosphatidic acid phosphatase that hydrolyzes phosphatidic acid (PA) to produce DAG. The activity of yeast lipin, Pah1, and hence the rate of DAG production is regulated by a protein phosphatase complex, comprised of two ER proteins, Nem1 and Spo7, which dephosphorylates and thereby activates Pah1. Like Fld1, Nem1 localizes to ER sites in proximity to LDs (**Fig. 1**). Seipin deficient cells accumulate numerous tiny LDs and a few supersized LDs, whereas lack of Nem1 results in a reduced number of LDs and the accumulation of neutral lipids in the ER membrane. FIT2, a widely conserved ER membrane protein, is expressed in all cells and is essential in higher eukaryotes. Previous work has shown that FIT2 depletion results in an arrest of LDs in the ER, and these LDs appear to emerge towards the ER lumen instead of the cytosol; this has been observed not only in yeast, but also in *C. elegance* and mice. The final factor known to affect LD formation is Pex30. This ER membrane protein has a reticulon homology domain and is thought to facilitate tubulation of the ER membrane at subdomains where LDs and pre-peroxisomal vesicles form. Previous work has shown that the deletion of Pex30 in an *fld1*Δ mutant background results in a strong synergistic growth defect, impaired LD biogenesis and the accumulation of neutral lipids in the ER.

In our recent study, we demonstrate that the biogenesis of LDs occurs at discrete ER subdomains defined by Fld1 and Nem1. Remarkably, localization of Fld1 and Nem1 at these ER subdomains is independent of each other, or of the presence of LDs, but both proteins are required together to create functional sites of LD biogenesis. At these Fld1/Nem1-containing ER subdomains the TAG-synthases, Lro1 or Dga1, get recruited as well as additional factors that promote LD biogenesis, including Yft2, Pex30, and the LD marker proteins Pet10 and Erg6. Proper localization of LD biogenesis in the ER is important, because in cells lacking either Fld1 or Nem1, TAG synthesis occurs ectopically throughout the ER. Ectopically formed LDs in *fld1*Δ mutant cells do not contain a complete set of LD proteins rendering them functionally impaired. Based on these findings we propose a model for a stepwise initiation of yeast LD biogenesis and an ordered recruitment of proteins to ensure a regulated biogenesis of functional LDs (**Fig. 2**).

**Figure 2:**
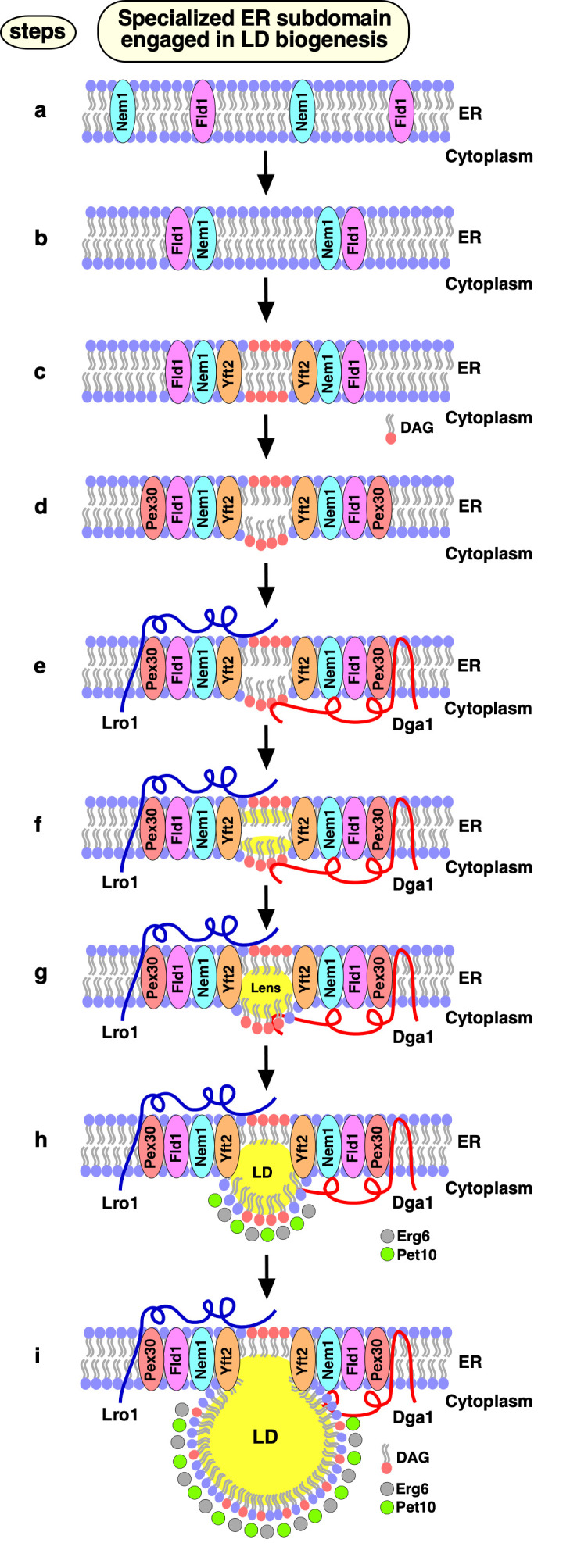
FIGURE 2: Model of LD biogenesis from specialized ER subdomains. **a)** Fld1 and Nem1 show punctate ER distribution in yeast mutants lacking LDs. **b)** Nem1 gets activated at sites where Fld1 and Nem1 colocalize (Fld1/Nem1 sites). **c)** Activation of Pah1 and production of DAG at these sites. DAG enrichment drives Yft2 recruitment at Fld1/Nem1 sites. **d)** Pex30 localizes to Fld1/Nem1/Yft2-containing ER subdomains, and deforms the ER bilayer. **e-g)** Sites containing Fld1/Nem1/Yft2/Pex30 become functional in recruiting TAG producing enzymes: Lro1 and Dga1 are recruited (e), resulting in neutral lipid synthesis **(f)**, and formation of neutral lipid lenses **(g)**. **h, i)** Lenses grow into nascent LDs that are recognized by bona fide LD marker proteins, such as Pet10 and Erg6, facilitating nascent LDs to emerge towards the cytoplasm (h) where they further grow in size and mature (i). Mature LDs acquire additional LD surface proteins and remain connected to the ER to promote bidirectional transport of proteins and lipids between the two compartments. This figure has been modified from Choudhary et al (2020). J Cell Biol; doi: 10.1083/jcb.201910177

Apart from the two TAG-synthases, Lro1 and Dga1, yeast expresses two STE synthases, Are1 and Are2. Mutant cells lacking all four of these neutral lipid biosynthetic enzymes (4ΔKO: *lro1*Δ *dga1*Δ *are1*Δ *are2*Δ) lack the capacity to produce storage lipids and hence have no LDs. All four of these enzymes are localized in the ER, but Dga1 can also localize to the surface of mature LDs. In 4ΔKO cells, Fld1 and Nem1 localize to discrete ER subdomains (**Fig. 2**, step a). What determines this punctate ER localization of Fld1 and Nem1 has not yet been established, but is likely mediated by protein and/or lipid cues. Most of the Nem1-containing ER puncta appear to be immobile in the ER; some Fld1 puncta show rapid mobility along the ER, while others appear to be stable. The sites where both Fld1 and Nem1 colocalize (Fld1/Nem1 sites) become functional for LD biogenesis (**Fig. 2**, step b). Induction of TAG synthesis results in an increased colocalization of Fld1 and Nem1, suggesting that the number of LD formation sites is coordinated with the levels of neutral lipids that are being produced. Nem1 activates Pah1 at Fld1/Nem1 sites, which in turn induces an increased local production of DAG (**Fig. 2**, step c). At this stage, Fld1 might prevent the outward diffusion of DAG into the ER bilayer. Consistent with this notion, DAG appears to be enriched at Fld1/Nem1 sites when cells were stimulated to produce LDs by the addition of oleic acid.

At this stage, Yft2 becomes enriched at Fld1/Nem1 sites, possibly due to its direct binding to DAG and/or TAG (**Fig. 2**, step c). Consistent with this, mammalian FIT2 has previously been shown to bind DAG and TAG *in vitro*. If Nem1 is missing from Fld1/Nem1 sites, Yft2 fails to get recruited, possibly because of limited DAG and/or TAG levels. In agreement with this hypothesis, overexpression of a hyperactive allele of *PAH1* (Pah1-7P), which bypasses the requirement for activation by Nem1, rescues the recruitment of Yft2 at Fld1 sites in cells lacking Nem1. After the association of Yft2 with Fld1/Nem1 sites, the membrane shaping protein Pex30 is recruited (**Fig. 2**, step d). Pex30 acts downstream of Fld1, Nem1 and Yft2, and its activity at these ER subdomains might promote deformation of the ER bilayer membrane, thereby helping to accommodate even more DAG and/or TAG. Consistent with this notion, previous studies have shown that an ER-anchored DAG sensor is enriched at sites containing Fld1, Nem1, Yft2 and Pex30.

Finally, the TAG-synthases, Lro1 or Dga1 get recruited at Fld1/Nem1/Yft2/Pex30 sites, catalyzing the conversion of DAG to TAG and hence formation of neutral lipid lenses and promoting their growth into nascent LDs (**Fig. 2**, steps e-h). In the absence of either Fld1 or Nem1, these TAG-synthases do not concentrate at discrete spots and instead localize throughout the ER, resulting in ectopic TAG synthesis. Lack of either Fld1 or Nem1 also results in mislocalization of Pex30 and Yft2. Hence, all four of these proteins Fld1/Nem1/Yft2/Pex30 appear to work together at ER subdomains to promote localized synthesis and packaging of neutral lipids. At the very last step, bona fide LD marker proteins, such as the perilipin homolog Pet10 and the ergosterol biosynthetic enzyme Erg6 get recruited to these nascent LDs, thereby stabilizing the LD surface and further promoting their growth and emergence towards the cytoplasm (**Fig. 2**, steps h, i).

In summary, our findings indicate that out of the vast interconnected ER network, ER subdomains defined by the colocalization of Fld1/Nem1/Yft2/Pex30, form sites dedicated to the biogenesis of LDs. Thus, LD formation is a spatially defined process and does not occur through a random coalescence of neutral lipids into lenses in the ER. Importantly, lack of any of the four key players impedes droplet formation and results in aberrant LDs. It will be interesting to see whether similar principles also apply to LD formation in animal cells and whether the human Nem1 orthologue, C-Terminal Domain Nuclear Envelope Phosphatase 1 (CTDNEP1, formerly Dullard), also requires colocalization with seipin to create functional ER subdomains.

